# PREDICTORS OF RESILIENCE AMONGST INFORMAL DEMENTIA CAREGIVERS

**DOI:** 10.1093/geroni/igad104.2551

**Published:** 2023-12-21

**Authors:** Kaitlin Grelle, Sarah Vacek, Kara Rayha, Willie Hale, Rebecca Deason

**Affiliations:** University of Texas at San Antonio, San Antonio, Texas, United States; University of Texas at San Antonio, San Antonio, Texas, United States; University of Texas at San Antonio, San Antonio, Texas, United States; University of Texas at San Antonio, San Antonio, Texas, United States; Texas State University, San Marcos, Texas, United States

## Abstract

Informal caregivers of People with Alzheimer’s Disease (PwAD) report greater levels of burden compared to non-caregivers, which is associated with an increase of impaired physical and mental health (Pinquart & Sorensen, 2003; Cheng, 2017; Zhou et al., 2021). Recent studies focusing on resilience (a person’s adaption to the physical and psychological demands of providing care) showed higher levels of resilience in caregivers was inversely related to depression, burden, and anxiety (Lopes Da Rosa et al., 2018; Zhou et al., 2021). With resilience being a protective factor against burden in caregivers, it is imperative to understand what psychological characteristics act as predictors of resilience. Participants (N = 59) completed questionnaires about resilience, mental health, and knowledge of Alzheimer’s disease. A classification and regression tree (CART) analysis was conducted to evaluate caregiver psychological characteristics as possible predictors of resilience. The analysis produced a tree with four terminal nodes, with one characterized by having scores greater than 152 for self-efficacy, followed by scores greater than 15 for knowledge of Alzheimer’s Disease. The overall model is 79.7% correct in predicted resilience, with a specificity of 95.7% and a sensitivity of 69.4%. These findings provide evidence that caregivers with greater confidence in their abilities are more likely to be resilient, especially for those with increased knowledge about Alzheimer’s disease (Choi et al., 2018; Kim & Bolkan, 2022). Interventions that focus on bolstering knowledge of Alzheimer’s disease and increasing self-efficacy may be critical in enhancing resilience in caregivers, in turn reducing depression and burden.

